# DNA Interaction Studies of a New Platinum(II) Complex Containing Different Aromatic Dinitrogen Ligands

**DOI:** 10.1155/2011/429241

**Published:** 2011-12-14

**Authors:** Nahid Shahabadi, Somaye Mohammadi, Robabeh Alizadeh

**Affiliations:** ^1^Department of Inorganic Chemistry, Faculty of Chemistry, Razi University, Kermanshah 74155, Iran; ^2^School of Chemistry, Damghan University, Damghan, Iran

## Abstract

A new mononuclear Pt(II) complex, [Pt(DMP)(DIP)]Cl_2_.H_2_O, in which DMP is 4,4-dimethyl-2,2-bipyridine and DIP is 4,7-diphenyl-1,10-phenantroline, has been synthesized and characterized by physicochemical and spectroscopic methods. The binding interaction of this complex with calf thymus DNA (CT-DNA) was investigated using fluorimetry, spectrophotometry, circular dichroism, viscosimetry and cyclic voltametry (CV). UV-VIS spectrum showed 4 nm bathochromic shift of the absorption band at 280 nm along with significant hypochromicity for the absorption band of the complex. The intrnisic binding constant (*K*_*b*_ = 2 × 10^4^ M^−1^) is more in keeping with intercalators and suggests this binding mode. The viscosity measurements showed that the complex-DNA interaction can be hydrophobic and confirm intercalation. Moreover, the complex induced detectable changes in the CD spectrum of CT-DNA. The fluorescence studies revealed that the probable quenching mechanism of fluorescence of the complex by CT-DNA is static quenching. The thermodynamic parameters (Δ*H* > 0 and Δ*S* > 0) showed that main interaction with hydrogenic forces occurred that is intercalation mode. Also, CV results confirm this mode because, with increasing the CT-DNA concentration, shift to higher potential was observed.

## 1. Introduction

During the recent years, only some platinum compounds such as cisplatin, carboplatin, and more recently nedaplatin and oxaliplatin have been known as antitumor compounds in clinical treatments; the research in this field is still increasing [1–5]. The original studies of metallointercalators centered on square planar platinum(II) complexes containing phenanthroline ligand [6, 7]. Octahedral transition metal complexes containing three bipyridine or phenanthroline ligands have been shown to be groove binders or possible partial intercalators [8, 9]. Concerning the noncovalent interactions between transition metal complexes and DNA, they can occur by intercalation, groove binding, or external electrostatic binding [10]. In general, metal complexes upon binding to DNA are stabilized through a series of weak interactions such as the π-stacking interactions of aromatic heterocyclic groups between the base pairs (intercalation), hydrogen bonding, and van der Waals interactions of functional groups bound along the groove of the DNA helix [11]. Platinum compounds are an important part of the first line of treatment for many types of cancers such as testicular and ovarian cancer. Cisplatin, cis-[PtCl_2_(NH_3_)_2_], and carboplatin, Pt(cbca)(NH_3_)_2_ with cbca = 1,1-cyclobutanedicarboxylate, has been in use clinically for more than 30 years. Recently, oxaliplatin, Pt(dach)(ox) where dach = R,R-1,2-diaminocycloexane and ox = oxalate, a third generation of Pt complexes have been approved for clinical use [12]. McFadyen et al. [13] synthesized a range of Pt(II) complexes containing substituted phenanthroline ligands and showed that the complex [Pt(en)(3,4,7,8-Me_4_phen)]Cl_2_ was two or three times more active than the corresponding phen-based complex [Pt(en)(phen)]Cl_2_, against murine leukemia cell lines. In this context, we report the results of an investigation of the interaction with calf thymus DNA of the complex [Pt(DMP)(DIP)]Cl_2_·H_2_O. The data were further compared with previous results [14] to determine the effect of metal on the DNA binding mode. Many techniques have been applied for investigation of the interaction of metal complexes with DNA. These include (i) molecular spectroscopy methods such as UV spectrophotometry [15], fluorescence [16], circular dichroism spectroscopy [17, 18], dynamic viscosity measurements [19, 20], and high-performance liquid chromatography [21]. Between these techniques, UV spectrophotometry, fluorescence method, dynamic viscosity measurements circular dichroism, and cyclic voltametry studies have been preferred because small molecule-DNA interaction may be experimentally monitored by changes in the intensity and position of the spectroscopic peak responses or changes in dynamic viscosity of DNA.

## 2. Experimental

All chemicals such as K_2_PtCl_4_, 4,7-dimethyl-1,10-phenanthroline, and 4,4-dimethyl-2,2-bypyridine, were purchased from Merck, and Tris-HCl and highly polymerized calf thymus DNA (CT-DNA) were purchased from Sigma Co.. Experiments were carried out in Tris-HCl buffer at pH 7.0. A solution of calf thymus DNA gave a ratio of UV absorbance at 260 and 280 nm more than 1.8, indicating that DNA was sufficiently free from protein [22]. The stock solution of CT-DNA was prepared by dissolving DNA in 10 mM of the Tris-HCl buffer at pH 7.0. The DNA concentration (monomer units) of the stock solution (1 × 10^−2^ M per nucleotide) was determined by UV spectrophotometer, in properly diluted samples, using the molar absorption coefficient 6600 M^−1^ cm^−1^ at 258 nm [23]. The stock solutions were stored at 4°C and used over no more than 4 days.

### 2.1. Synthesis of the [Pt(DMP)(DIP)]Cl_2_·H_2_O Complex

**Figure 1:**
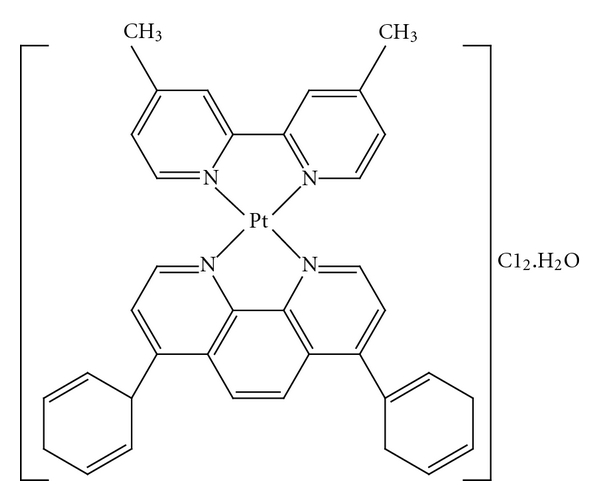
The structure of [Pt(DMP)(DIP)]Cl_2_·H_2_O complex.

[Pt(DMP)(DIP)]Cl_2_·H_2_O (Figure 1) was synthesized by two steps. In the first step the platinum precursor, K_2_PtCl_4_, the (0.05 g, 0.12 mmol) was dissolved in 6 mL of water and a solution of 4,4-dimethyl-2,2-bipyridine (0.022, 0.12 mmol) was added dropwise. The mixture was stirred overnight at 50°C. A yellow precipitate was formed, which was filtered off and washed with ice-cold water and acetone. Subsequently in second step the prepared precipitate in first step (0.03 g, 0.1 mmol) was dissolved in 4 mL methanol and a solution of 4,7-diphenyl-1,10-phenantroline (0.027 g, 0.1 mmol) in 4 mL methanol was added dropwise. The last solution was refluxed in 180°C for 4 h. A green precipitate was formed, which was filtered off and washed with ice-cold water and diethylether. Yield: 36%. Elemental analysis for PtC_36_H_28_N_4_Cl_2_H_2_O: found: C, 53.2; H, 4.1; N, 6.9%; calc.: C, 54.1; H, 3.6; N, 7.0%; FT-IR (KBr, cm^−1^): 3050–3236 [**ν** (C–H)], 1230 [**ν** (C–N)], 1560 [**ν** (C=N)], 532 [**ν** (Pt–N)], 3400–3500 [*ν* (H_2_O)].

### 2.2. Instrumentation

^1^H NMR spectra were recorded with using a Bruker Avance DPX200 MHz (4.7 T) spectrometer using d6-DMSO as solvent. The elemental analysis was performed using Heraeus CHN elemental analyzer. Absorbance spectra were recorded using a hp spectrophotometer (agilent 8453) equipped with a thermostated bath (Huberpolysat cc1). Absorption titration experiments were carried out by keeping the concentration of DNA constant (5 × 10^−5^ M) while varying the complex concentration from 5 × 10^−5^ M to 4 × 10^−4^ M (ri = [DNA]/[complex] = 0–4).

**Figure 2:**
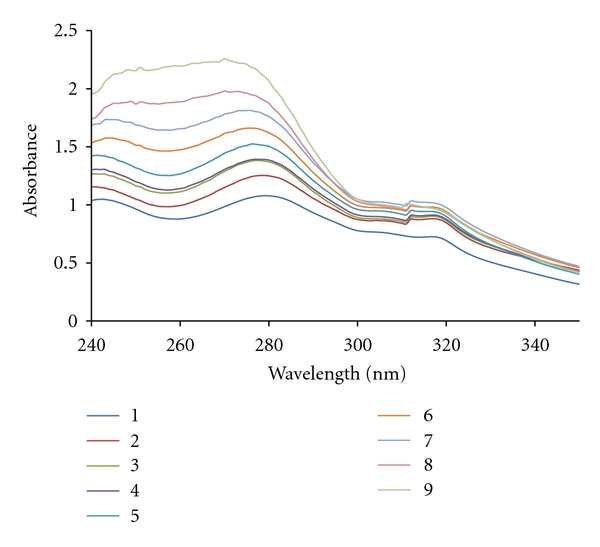
Absorption spectra of DNA (5 × 10^−5^ M) in the absence and in the presence of increasing amounts of platinum complex (ri = [DNA]/[complex]).

Absorbance values were recorded after each successive addition of DNA solution and equilibration (ca. 10 min) (Figure 2). The data were then fitted to (1) to obtain intrinsic binding constant, *K*_*b*_ [24]:

(1) $$\frac{\left[\text{DNA}\right]}{\left(\varepsilon_{a}-\varepsilon_{f}\right)}=\frac{\left[\text{DNA}\right]}{\left(\varepsilon_{b}-\varepsilon_{f}\right)}+\frac{1}{K_{b}\left(\varepsilon_{b}-\varepsilon_{f}\right)},$$

where *ε*_*a*_, *ε*_*f*_, and *ε*_*b*_ are the apparent, free and bound complex extinction coefficients, respectively. In particular, *ε*_*f*_ was determined by a calibration curve of the isolated metal complex in aqueous solution; following the Beer's law, *ε*_*a*_ was determined as the ratio between the measured absorbance and the Pt(II) complex concentration, *A*obs/[Pt]. A plot of [DNA]/(*ε*_*a*_ − *ε*_*f*_) versus [DNA] gave a slope of 1/(*ε*_*b*_ − *ε*_*f*_) and a *Y *intercept equal to 1/*K*_*b*_(*ε*_*b*_ − *ε*_*f*_); *K*_*b*_ is the ratio of the slope to the *Y* intercept. CD measurements were recorded on a JASCO (J-810) spectropolarimeter, keeping the concentration of DNA constant (5 × 10^−5^ M) while varying the complex concentration (ri = [complex]/[DNA] = ri = 0, 0.4, 0.5).

Viscosity measurements were made using a viscosimeter (SCHOT AVS 450) maintained at 25.0 ± 0.5°C using a constant temperature bath. The DNA concentration was fixed at 5 × 10^−5^ M, and flow time was measured with a digital stopwatch. The mean values of three measurements were used to evaluate the viscosity *η* of the samples. The values for relative specific viscosity (*η*/*η*_0_)^1/3^, where *η*_0_ and *η* are the specific viscosity contributions of DNA in the absence (*η*_0_) and in the presence of the complex (*η*), were plotted against ri (ri =[complex]/[DNA] = 0.0–2.5).

All fluorescence measurements were carried out with a JASCO spectrofluorometer (FP6200) by keeping the concentration of complex constant while varying the DNA concentration from 0 to 36 × 10^−5^ M (ri = [DNA]/[complex] = 0.0, 0.5, 1.0, 1.5, 3, 3.5) at three different temperatures (288, 298, 308 K).

The cyclic voltammetric measurements were performed using an AUTOLAB model (PG STAT C), with a three-electrode system: a 0.10 cm diameter glassy carbon (GC) disc as working electrode, an Ag/AgCl electrode as reference electrode, and a Pt wire as counter electrode. Electrochemical experiments were carried out in a 25-mL voltammetric cell at room temperature. All potentials are referred to the Ag/AgCl reference. Their surfaces were freshly polished with 0.05 mm alumina prior to each experiment and were rinsed using double-distilled water between each polishing step. The supporting electrolyte was 0.01 M of Tris-HCl buffer solution (pH 7.4) which was prepared with double-distilled water. Before experiments, the solution was deaerated via purging with pure nitrogen gas for 1 min, and, during measurements a stream of nitrogen was passed over the solution. The current-potential curves and experimental data were recorded on software GPES [25].

## 3. Results and Discussion

### 3.1. Synthesis and Characterization of [Pt(DMP)(DIP)]Cl_2_·H_2_O Complex

The mixed ligand platinum(II) complex of 4,4-dimethyl-2,2-bipyridine (DMP) and 4,7-diphenyl-1,10-phenantroline (DIP) has been synthesized in aqueous methanol solution using K_2_PtCl_4_. The complex conforms to the formula [Pt(DMP)(DIP)]Cl_2_·H_2_O, determined on the basis of elemental analysis.

The IR spectrum of the complex was characterized by the appearance of a band at 532 cm^−1^ due to the [*ν* (Pt–N)]. The coordination of the nitrogen atoms is confirmed with the presence of this band. However, the broad band at 3400–3500 cm^−1^ is assigned to [*ν*(H_2_O)]. Other bands are at 3050–3236 [**ν** (C–H)], 1230 [**ν** (C–N)], and 1560 [**ν** (C=N)], respectively.

**^1^H NMR (d6-DMSO)**

In the aromatic region the signals at *δ* = 8.3, *δ* = 8.1 and *δ* = 7.1 ppm were assigned to protons of DMP ligands and the signals at *δ* = 8.9, *δ* = 7.6, *δ* = 7.5, and *δ* = 7.3 were assigned to the protons of DIP ligand. The protons of DMP and DIP ligands are seen to be shifted (*≈*−0.2 ppm) with corresponding free ligand and suggesting complexation.

### 3.2. UV-VIS Spectroscopy

Absorption spectroscopy is one of the most useful techniques to study the binding of any drug to DNA. The extent of hypochromism generally indicates the intercalative binding strength [26]. The hypochromicity characteristic of intercalation has usually been attributed to the interaction between the electronic states of the compound and those of the DNA bases [27], while the red shift has been associated with the decrease in the energy gap between the HOMO and LUMO molecular orbitals after binding of the complex to DNA [28]. Hyperchromism has been observed for the interaction of many drugs with DNA [29]. The hyperchromic effect might be ascribed to external contact (electrostatic binding [30]) or to partial uncoiling of the helix structure of DNA, exposing more bases of the DNA [31]. We have observed 4 nm bathochromic shift of the absorption band at 280 nm along with significant hypochromicity for the absorption band of the complex. This observation gives a good evidence of the intercalation of Pt(II) complex through the stacking and interaction of the aromatic rings of the ligands and the base pairs of DNA [32]. Additionally, based on the absorbance values obtained in the spectroscopic titration, the [Pt(DMP)(DIP)]Cl_2_·H_2_O complex-DNA binding constant, *K*_*b*_, was calculated as described in the experimental procedure. The value obtained for *K*_*b*_ was 2.0 ± 0.2 × 10^4^ M^−1^. The values of *K*_*b*_ described in the literature for classical intercalators (ethidium-DNA 7 × 10^7^ M^−1^ [33] and proflavin-DNA 4.1 × 10^5^ M^−1^ [34]) are at least 30 or 20 orders of magnitude higher than that obtained for this Pt(II) complex. This result suggests that intercalation between the base pairs is not the main mode of interaction of the Pt(II) complex with DNA. In contrast, the value of *K*_*b*_ is ten orders of magnitude higher than the *K*_*b*_ values which were found for compounds with the mode of groove binding like Cr(II) complexes [35] or tris (1,10-phen) ruthenium(II) to DNA [36]. These results confirm that the Pt(II) complex strongly interact with DNA, and the *K*_*b*_ value obtained for this complex is of the same order of magnitude of that determined in analogues condition for ZnL^+2^ (*K*_*b*_ = 7.36 ± 0.01 × 10^4^ M^−1^) which considered as an intercalating complex [37].

#### 3.2.1. Circular Dichroism Spectroscopy

**Figure 3:**
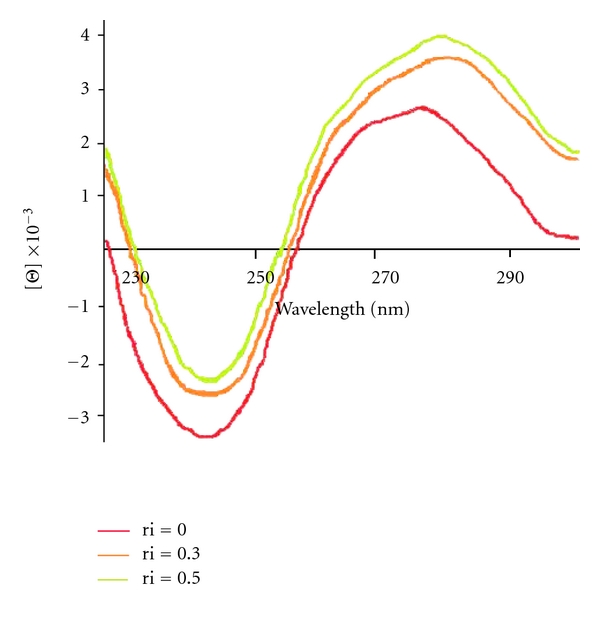
Circular dichroism spectra of CT-DNA (5 × 10^−5^ M) in the presence of increasing amounts of Pt(II) complex at the following stoichiometric ratios (ri = [complex]/[DNA] = 0.0, 0.4, 0.5).

To establish in more detail whether binding of the complex brings about any significant conformational change of the DNA double helix, circular dichroism (CD) spectra of CT-DNA were recorded at increasing complex/CT-DNA ratios. The observed CD spectrum of natural calf thymus DNA consists of a positive band at 275 nm due to base stacking and a negative band at 245 nm due to helicity, which is characteristic of DNA in right-handed B form (Figure 3) [38]. The effect of the complex on the conformation of the secondary structure of DNA was studied by keeping the concentration of CT-DNA at 5 × 10^−5^ M while varying the concentration of complex in a buffer solution of 10 mM of Tris (ri = 0, 0.4, 0.5). The spectrum of the CT-DNA and those with the additives were monitored from 215 to 800 nm. As shown in Figure 3, the CD spectrum of DNA exhibits a positive absorption at 282 nm due to the base stacking and a negative band at 240 nm due to the helicity of B-DNA. In the presence of the platinum(II) complex, the intensity of the positive peak increased and the intensity of the negative peak decreased. The changes in the CD spectra in the presence of the complex show stabilization of the right-handed B form of CT-DNA (Figure 3).

#### 3.2.2. Viscosity Measurements

**Figure 4:**
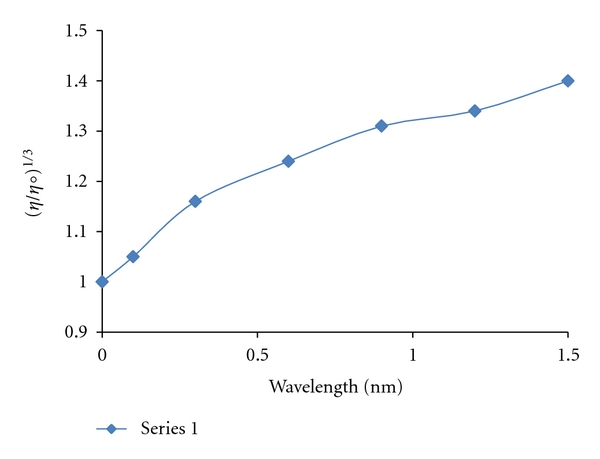
Effect of increasing amounts of Pt(II) complex on the viscosity of CT-DNA (5 × 10^−5^ M) in 10 mM Tris-HCl buffer.

The viscosity measurements of CT-DNA are regarded as the least ambiguous and the most critical tests of a binding model in solution in the absence of crystallographic structural data. The values of relative specific viscosity (*η*/*η*_0_)^1/3^ (where *η*_0_ and *η* are the specific viscosity contributions of DNA in the absence and in the presence of the Pt complex, resp.) were plotted against 1/*R *(*R *= [DNA]/[complex]) (Figure 4). It is known that the groove binder like Hoechst 33258 does not cause an increase in the axial length of the DNA [39, 40] and, therefore, did not alter the relative viscosity. In contrast, cisplatin which is known to link DNA through covalent binding, shortening the axial length of the double helix [41], caused a decrease in the relative viscosity of the solution. Partial intercalators also reduce the axial length observed as a reduction in relative viscosity, whereas the classical organic intercalators such as ethidium bromide increased the axial length of the DNA, and it becomes more rigid [39, 40] resulting in an increase in the relative viscosity. Results confirm the sensitivity of viscosity measurements to the different modes of DNA binding. In this study, it was observed that increasing the platinum complex concentration leaded to an increase of the DNA viscosity. Thus, we may deduce that the [Pt(DMP)(DIP)]Cl_2_·H_2_O complex certainly is a DNA intercalator. Since the interaction of this platinum(II) complex with DNA can make DNA longer, we would expect that the relative viscosity of DNA increases with a slope between 0 and 0.94 (a value measured for ethidium bromide [42] if the intercalation of the Pt(II) complex was either only one interaction mode or much stronger than other interaction(s) (Figure 6). But, in this study, the relative viscosity of DNA increases with a slope of 0.38 (Figure 4) and it is reasonably believed that may be other interaction(s) between DNA and the Pt(II) complex occurred, and is reasonable for the decrease of the slope. In addition, it should be noted that the DNA binding constant measured for this Pt(II) complex is lower than those determined for ethidium bromide. Therefore, the greater increase in viscosity observed for ethidium bromide compared to the Pt(II) complex is likely due to the lower binding constant of the latter to DNA. These results clearly show the importance of using several techniques to ascertain intercalation.

#### 3.2.3. Fluorescence Studies

**Figure 5:**
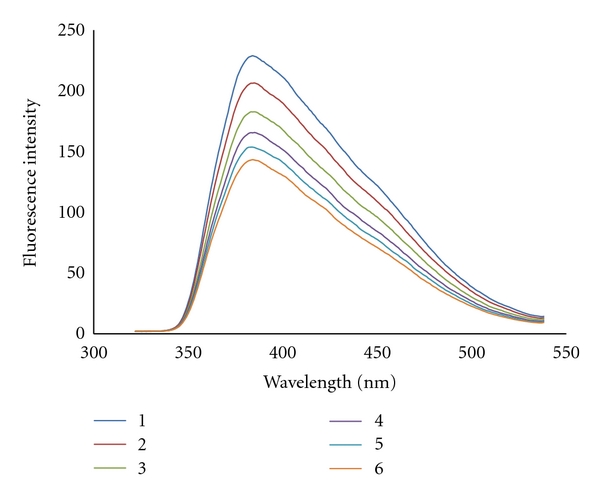
Fluorescence spectra of Pt(II) complex in the several concentrations of CT-DNA.

**Figure 6:**
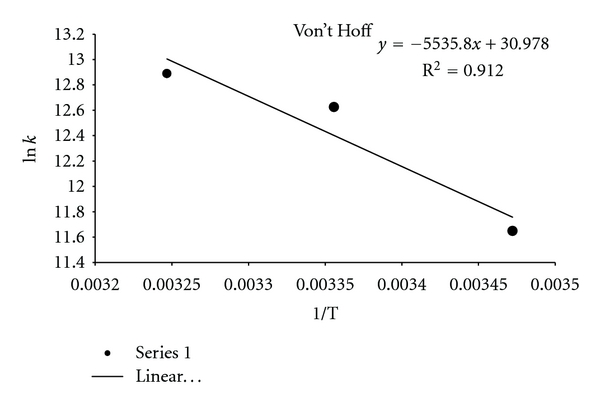
Van't Hoff plot for the interaction of the Pt(II) complex and CT-DNA at pH 7.2.

To further investigate the interaction mode between the complex and CT-DNA, fluorescence titration experiments was performed. The complex emits luminescence in Tris-HCl buffer with maximum wavelengths of about 386 nm.  Figure 5 shows the emission spectra of the complex in the absence and presence of varying amounts of CT-DNA. In the emission spectra for the complex, with increasing CT-DNA concentration the emission intensity is decreased due to self-stacking of some free bases in the compound along the DNA surface [43]. Quenching can occur by different mechanisms, which are usually classified as dynamic and static quenching. Dynamic quenching refers to a process in which the fluorophore and the quencher come into contact during the transient existence of the exited state, while static quenching refers to fluorophore-quencher complex formation. In general, dynamic and static quenching can be distinguished by their differing dependence on temperature and excited state lifetime. Since in both cases the fluorescence intensity is related to the concentration of the quencher, the quenched fluorophore can serve as an indicator for the quenching agent [44].

Fluorescence quenching is described by the Stern-Volmer equation:

(2) $$\frac{F_{0}}{F}=1+K_{q}\tau_{0}\left[Q\right]=1+K_{\text{SV}}\left[Q\right],$$

where *F*_0_ and *F* represent the fluorescence intensities in the absence and in the presence of quencher, respectively. *K*_*q*_ is the fluorophore quenching rate constant, *K*_SV_ is quenching constant, *τ*_0_ is the lifetime of the fluorophore in the absence of a quencher (*τ*_0_ = 10^−8^), and [*Q*] is the concentration of quencher [45]. The results in Table 1 indicate that the probable quenching mechanism of this complex by CT-DNA involves static quenching, because *K*_SV_ decreases with increasing temperature [46]. 

### 3.3. Binding Constant and the Number of Binding Sites

If it is assumed that there are similar and independent binding sites in the biomolecule, the binding constant (*K*_*f*_) and the number of binding sites (*n*) can be determined according to the method described by Hu et al. [47], using the following equation:

(3) $$\text{Log}\left(\frac{F_{0}-F}{F}\right)=\text{Log}\;K_{f}+n\;\text{Log}\left[Q\right].$$

Here, *F*_0_ and *F* are the fluorescence intensities of the fluorophore in the absence and presence of different concentrations of CT-DNA, respectively. The values of *K*_*f*_ and *n* were found to be 1.14 × 10^5^ M^−1^ and 1, respectively, (Table 2).

#### 3.3.1. Binding Mode between the Complex and DNA

According to the thermodynamic data, interpreted as follows, the model of interaction between a drug and biomolecule can be [48] (1) Δ*H* < 0 and Δ*S* < 0, hydrophobic forces; (2) Δ*H* > 0 and Δ*S* > 0, van der Waals interactions and hydrogen bonds; (3) Δ*H* > 0 and Δ*S* < 0, electrostatic interactions [49]. In order to elucidate the interaction of our complex with DNA, the thermodynamic parameters were calculated. The plot of ln⁡*K* versus 1/*T* (Figure 4; (4)) allows the determination of Δ*H* and Δ*S*. If the temperature does not vary significantly, the enthalpy change can be regarded as a constant. Based on the binding constants at different temperatures, the free energy change can be estimated (Table 3; (5)) by the following equations

(4) $$\begin{array}{c} \text{ Ln}\;K=-\frac{\Delta H}{RT}+\frac{\Delta S}{RT}, \end{array}$$

(5) $$\begin{array}{c} \Delta G=\Delta H-T\Delta S=-RT\text{Ln}K, \end{array}$$

where *K* is the Stern-Volmer quenching constant at the corresponding temperatures and *R* is the gas constant. When we apply this analysis to the binding of the complex with CT-DNA, we find that Δ*H* > 0 and Δ*S* > 0. Therefore, van der Waals interactions are probably the main forces in the binding of the investigated complex to CT-DNA.

### 3.4. Electrochemical Behaviour of the Complex in the Absence and Presence of CT-DNA

**Figure 7:**
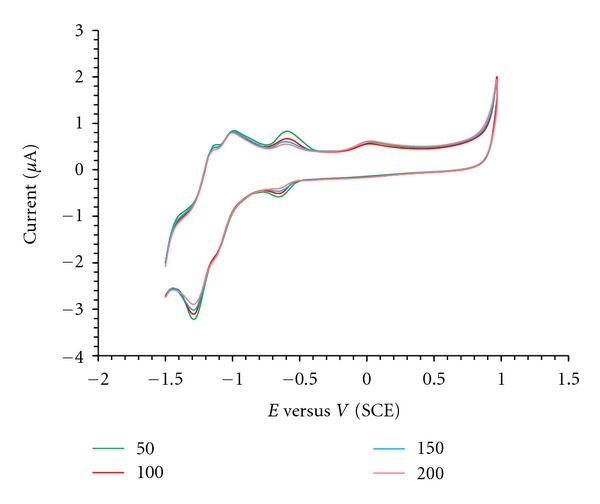
Cyclic voltametry for the Pt (II) complex (10^−3^ mol/L) at the presence of different concentrations of DNA.

Recently, the electrochemical techniques extensively were used as a simple and rapid method to study DNA interaction with different compounds. The electrochemical behaviour of platinum is well known and was strongly influenced by the electrode material. A well-defined and sensitive peak was observed from the solutions of the complex with a GC electrode rather than the Pt one. Therefore, a GC electrode was used in this investigation. When CT-DNA is added to a solution of the complex, both the anodic and cathodic peak current heights of the complex decreased in the same manner of increasing additions of DNA, (Figure 7). Also during DNA addition, the anodic peak potential (Epa), cathodic peak potential (Epc), and *E*_1/2_ (calculated as the average of Epc and Epa) all showed positive shifts. These positive shifts are considered as evidences for intercalation of the complex into the DNA, because this kind of interaction is due to hydrophobic interaction. From the other point of view, if a molecule binds electrostatically to the negatively charged deoxyribose-phosphate backbone of DNA, negative peak potential shifts should be detected. Therefore, the positive shift in the CV peak potentials of complex is indicative of intercalative binding mode of the complex with DNA [25].

## 4. Conclusion

In this paper, we investigate synthesis and characterization of new platinum(II) complex, and binding with CT-DNA was studied. The results suggest that the complex binds to DNA via intercalation mode. Spectrophotometry showed 4 nm bathochromic shift of the absorption band at 280 nm along with significant hypochromicity for the absorption band of the complex. The intristic binding constant (*K*_*b*_ = 2 × 10^4^ M^−1^) is more in keeping with intercalators and suggest this binding mode. The viscosity measurement showed that the complex-DNA interaction can be hydrophobic and confirm intercalation. Moreover, the complex induced detectable changes in the CD spectrum of CT-DNA. The fluorescence studies revealed that the probable quenching mechanism of fluorescence of the complex by CT-DNA is static quenching. The thermodynamic parameters (Δ*H* > 0 and Δ*S* > 0) showed that van der Waals interactions occurred that is intercalation mode. Also, CV results confirm this mode because, with increasing CT-DNA concentration, shift to higher potential was observed.

## Figures and Tables

**Table 1:** The quenching constants of the Pt(II) complex by CT-DNA at different temperatures.

*T* (K)	*R* ^2^	*K*_SV_ (Lmol^−1^) × 10^5^	*K*_*q*_ (Lmol^−1^) × 10^13^
288	0.9946	3.53	3.53
298	0.9981	5.42	5.42
308	0.9965	10.34	10.34

**Table 2:** Binding constants (*K*_*f*_) and number of binding sites (*n*) of the complex-DNA system.

*T* (K)	*n*	Log *K*_*f*_	*K*_*f*_	*R* ^2^
288	0.919	5.06	1.14 × 10^5^	0.9979
298	0.958	5.48	3.04 × 10^5^	0.9993
308	0.931	5.60	3.96 × 10^5^	0.9991

**Table 3:** Thermodynamic parameters for the binding of the Pt(II) complex to CT-DNA.

*T* (K)	Δ*G* (KJ mol^−1^)	Δ*H* (KJ mol^−1^)	Δ*S* (J mol^−1^ K^−1^)
288	−28.15	46.03	257.55
298	−30.73	46.03	257.55
308	−33.30	46.03	257.55
